# Characterization of the complete chloroplast genome of a medicinal plant, *Wikstroemia indica* (Thymelaeaceae)

**DOI:** 10.1080/23802359.2019.1696249

**Published:** 2019-12-12

**Authors:** Shao-Juan Qian, Yong-Hong Zhang

**Affiliations:** School of Life Sciences, Yunnan Normal University, Kunming, China

**Keywords:** *Wikstroemia indica*, chloroplast genome, medicinal plant, phylogenetic analysis

## Abstract

The complete chloroplast genome of *Wikstroemia indica*, a medicinal plant with a variety of medicinal functions in treatment for arthritis, whooping cough, cancer, and bacillosis, was generated by de novo assembly using whole genome next-generation sequencing. The plastome was a quadripartite circular with 151,731 bp in length; containing a large single-copy (LSC) region of 86,523 bp and a small single-copy (SSC) region of 12,384 bp; separated by 2 inverted repeat (IR) regions of 26,403 bp each. The chloroplast genome contained 124 genes, including 79 protein-coding genes, 37 tRNA genes, and 8 rRNA genes. The GC content in the whole cp genome, LSC region, SSC region, and IR region were 37.4, 34.9, 32.4, and 42.6%, respectively. The phylogenetic tree indicated that *W. indica* has a close relationship with *Stellera chamaejasme* with 100% support.

*Wikstroemia indica* (L.) C. A. Mey., a toxic and medicinal shrub distributed in Vietnam, India, and China growing in open forest or on stone mountain, has been used as a traditional Chinese medicine named ‘Liao Ge Wang’ with a variety of medicinal functions in arthritis, whooping cough, cancer, and bacillosis (Wang and Gilbert 2007, Chen et al. 2016). Previous studies on *W. indica* were focused on the determination of effective active ingredients (Kato et al. 2014), extraction methods (Sun et al. 2012, Chang et al. 2017), pharmacological analysis, and clinical application (Chen et al. 2016), and lack of molecular research. In this study, we reported the complete cp genome of *W. indica* to provide genomic resource for molecular research and phylogenetic analysis.

Fresh leaves were collected from a healthy *W. indica* plant at Debao county (23°04′03″N, 106°25′19″E), the Guangxi Zhuang Autonomous Region, China. The voucher specimens (ZGG-001) were deposited in the Herbarium of Yunnan Normal University. A sequence library was constructed and sequencing was performed using the Illumina HiSeq 2500-PE150 platform. (Illumina, San Diego, CA, USA). All raw reads were filtered using NGS QC Toolkit_v2.3.3 with default parameters to obtain clean reads (Patel and Jain 2012). The plastome was de novo assembled using NOVOPlasty (Dierckxsens et al. 2017). The cp genome sequence was annotated using Geneious 9.1 (Kearse et al. 2012).

The complete chloroplast genome of *W. indica* (Genbank accession no.: MN453832) was a quadripartite circular with 151,731 bp in length, including a large single-copy (LSC) region of 86,532 bp, and small single-copy (SSC) region of 12,384 bp, separated by 2 inverted repeat (IR) regions of 26,403 bp, respectively. The GC content in whole cp genome, LSC region, SSC region, and IR region were 37.4, 34.9, 32.4, and 42.6%, respectively. The chloroplast genome contained 79 protein-coding genes (71 PCG species), 37 transfer RNA genes (29 tRNA species), and 8 rRNA genes (4 rRNA species). A total of 95 SSRs were discovered by the online software IMEx (Mudunuri and Nagarajaram 2007). Among them, the numbers of mono-, di-, tri-, tetra- and penta-nucleotides SSRs are 74, 11, 3, 5 and 1, respectively.

The published chloroplast genomes from Malvales, including 7 species from Thymelaeaceae and 9 species from Malvaceae, were used to identify the phylogenetic position of *W. indica* with three species from Brassicales as outgroups. All sequences were aligned by MAFFT 7.308. (Katoh and Standley 2013) and the maximum likelihood (ML) tree was reconstructed by RAxML 8.2.11 (Stamatakis et al. 2008) with the nucleotide substitution model of GTR + G. Bootstrap values were calculated from 1000 replicate analysis. Phylogenetic analysis revealed that all sampled species of Thymelaeaceae formed a monophyletic clade with 100% support. Within Thymelaeaceae, *W. indica* has a close relationship with *Stellera chamaejasme* with 100% support (Figure 1). The complete chloroplast genome of *W. indica* will provide a useful resource for the in-depth molecular study of this species as well as for the phylogenetic studies of Thymelaeaceae.

**Figure 1.**
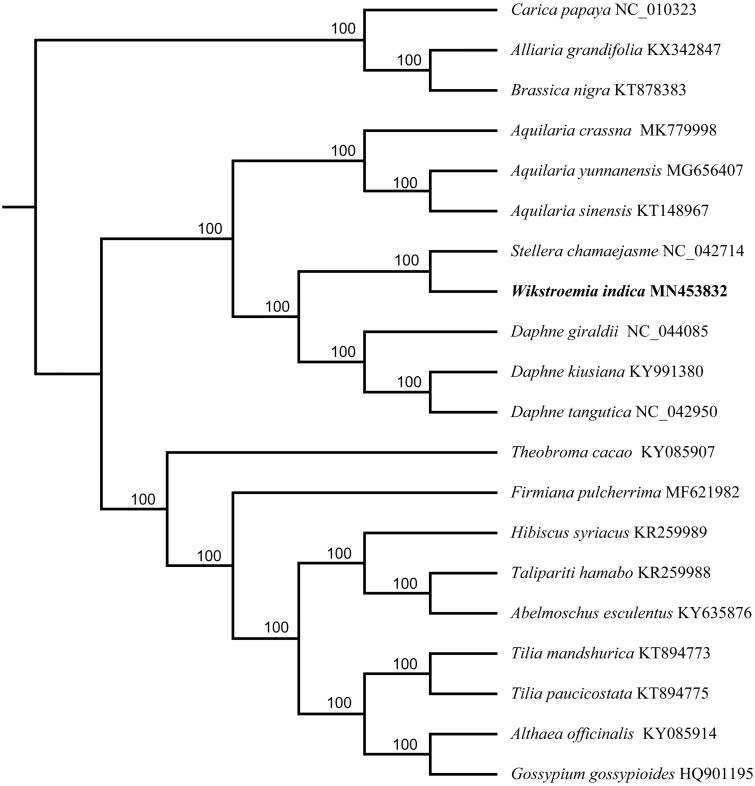
Maximum-likelihood (ML) tree of *W. indica* and its related relatives based on the complete chloroplast genome sequences. Bootstrap values from 1000 replicates were shown next to the nodes.
